# Left ventricular transthyretin amyloid load and apical sparing in patients with newly confirmed transthyretin amyloid cardiomyopathy

**DOI:** 10.1002/ejhf.70077

**Published:** 2025-10-30

**Authors:** Thomas Krammer, Maria J. Baier, Vanessa Lutz, Anna‐Christina Hübner, Tilman Zschiedrich, David Lukas, Christian Le Phu, Matthias Wolf, Claire Maassen, Michael Wester, Stefan Neef, Christian Schach, Can‐Martin Sag, Katja Evert, Michael Paulus, Christine Meindl, Maria Tafelmeier, Kurt Debl, Lars S. Maier, Stefan Wagner, Christoph Röcken, Julian Mustroph

**Affiliations:** ^1^ Department of Internal Medicine II University Medical Center Regensburg Regensburg Germany; ^2^ Institute for Pathology University of Regensburg Regensburg Germany; ^3^ Institute for Pathology University of Kiel Kiel Germany; ^4^ Department of Pharmacology University of Regensburg Regensburg Germany

**Keywords:** Transthyretin amyloid cardiomyopathy, ATTR, Amyloidosis, Apical sparing, Amyloid load, Perugini score

## Abstract

**Aims:**

Transthyretin amyloid cardiomyopathy (ATTR‐CM) is marked by deposition of transthyretin amyloid in the myocardium. Patients present with symptoms of heart failure, left ventricular (LV) hypertrophy, diastolic dysfunction, and arrhythmias. Echocardiographic apical sparing, quantified via the relative apical sparing (RELAPS) pattern, is a hallmark imaging feature but its histopathological and clinical implications remain uncertain. This study investigated the association between apical sparing, myocardial amyloid load, and clinical phenotypes in newly diagnosed ATTR‐CM.

**Methods and results:**

We prospectively enrolled 61 patients undergoing LV endomyocardial biopsy for suspected amyloidosis between May 2022 and May 2024. After histological confirmation, 56 patients with wild‐type ATTR‐CM were included. LV amyloid load was quantified from Congo red‐stained endomyocardial biopsies. Echocardiographic parameters including global longitudinal strain (GLS) and RELAPS were assessed peri‐interventionally. Clinical, laboratory, and imaging features were compared between patients with and without RELAPS. Patients with RELAPS had significantly higher LV amyloid load than those without. RELAPS was associated with elevated N‐terminal pro‐B‐type natriuretic peptide levels, higher Perugini scores, lower GLS and atrial strain. No differences between patients with and without RELAPS were found regarding age or wall thickness. RELAPS correlated with markers of disease severity and atrial remodelling. The Perugini scores failed to distinguish intermediate levels of myocardial amyloid content in 71.1% of cases.

**Conclusions:**

Apical sparing reflects advanced myocardial involvement in ATTR‐CM and correlates with increased amyloid load and biomarkers in a large endomyocardial biopsy collective. RELAPS, together with histological amyloid quantification, offers valuable insights for risk stratification and may guide therapeutic intervention in this progressive disease.

## Introduction

Transthyretin amyloid cardiomyopathy (ATTR‐CM), once considered a rare disease of the elderly, has recently taken the centre stage in cardiology due to a rapidly increasing incidence,^1^ linked to better awareness of the disease, ageing populations, and novel effective therapeutic options such as transthyretin (TTR) stabilizers (e.g. acoramidis, tafamidis) and RNA interference (e.g. vutrisiran).

Physiologically, TTR forms a stable tetramer in the blood. Structural destabilization can lead to tetramer dissociation, monomer misfolding, and aggregation into amyloid fibrils. Deposits of the wild‐type form of TTR (i.e. in patients without TTR gene mutations) occur most often in the myocardium. In hereditary TTR amyloidosis, the clinical phenotype varies depending on the location of amyloid deposition; involvement of soft tissues may lead to compressive neuropathies, such as carpal tunnel syndrome.^2^, ^3^, ^4^ In patients with wild‐type ATTR‐CM, a progressive heart failure phenotype including left ventricular (LV) hypertrophy, diastolic dysfunction, and arrhythmias, is a hallmark of the disease. The European Society of Cardiology guidelines for the diagnosis and treatment of cardiomyopathies recommend echocardiography and magnetic resonance imaging (MRI) for most patients with a hypertrophic phenotype.^5^ In practice, the limited availability of MRI scans often prompts a direct ‘bone tracer’ scintigraphy scan in patients with signs of ATTR‐CM (such as hypertrophy) and additional red flags.^6^ In theory, a positive scintigraphy offers a high sensitivity for cardiac TTR deposits (with acceptable specificity^7^), sufficient for the diagnosis of ATTR‐CM.^6^ However false positives and negatives occur^6^ and the visually assessed Perugini score^6^ is susceptible to interobserver variability as demonstrated in the PRACTICA study.^8^ Also, elevated serum free light chains or the presence of monoclonal proteins in the immunofixation in patients with suspected amyloid cardiomyopathy require exclusion of cardiac light chain (AL) amyloidosis, which is not possible non‐invasively. Therefore, endomyocardial biopsy remains the diagnostic gold standard and the presence of TTR in cardiac biopsies from patients with a clinical phenotype confirms ATTR‐CM.^6^ In contrast to the qualitative verification of amyloid presence as a proven diagnostic tool for ATTR‐CM, the relevance of the amyloid quantity or ‘cardiac amyloid load’ in endomyocardial biopsies has only been investigated for AL‐amyloidosis.^9^ Moreover, studies trying to establish links between the presence of amyloid and echocardiographic features of ATTR‐CM suffer from low number of patients (e.g. four deceased ATTR‐CM patients with available echocardiography and cardiac autopsy^10^).

Nevertheless, echocardiographic strain analysis has emerged as a key tool for the investigation of ATTR‐CM^6^ and low global longitudinal strain (GLS) is associated with adverse outcome.^11^ A characteristic echocardiographic finding in ATTR‐CM is the apical sparing phenomenon, a strain pattern wherein the apex exhibits better (i.e. more negative) longitudinal strain values compared to the basal segments of the left ventricle. While apical sparing can be assessed by ‘eye‐balling’, a less subjective measure is the relative apical sparing (RELAPS) pattern, which can be calculated by the formula average apical longitudinal strain/(average basal longitudinal strain + average midventricular longitudinal strain). Values >1 indicate a positive RELAPS.^12^, ^13^ RELAPS in echocardiography is sensitive and relatively specific for the diagnosis of cardiac amyloidosis^14^ and is associated with adverse outcomes, including death.^15^ The finding of RELAPS is suggestive of better contractility in the apex, but its underlying pathophysiological mechanism is undefined (a small post‐mortem series investigating TTR load and apical sparing only included two patients with myocardial strain analysis^10^). In this observational study, we prospectively included patients destined to undergo LV endomyocardial biopsy for the diagnosis of cardiac amyloidosis and—in addition to standard histology—analysed LV amyloid load. Periprocedurally, patients underwent echocardiography to assess LV function and to determine GLS values as well as the presence of RELAPS. Moreover, we set out to correlate the Perugini values of the patients with scintigraphy scans with our amyloid load analysis (*Graphical Abstract*). To our knowledge, our study is the largest biopsy collective of patients undergoing endomyocardial biopsy to test for ATTR‐CM and provides clinically relevant insights into its pathophysiology at the time of diagnosis.

## Methods

A detailed methodological description can be found in the online supplementary material.

### Study protocol

In this observational study, after informed consent, we prospectively enrolled 61 patients presenting to our clinic with high probability of cardiac amyloidosis between May 2022 and May 2024. The study was approved by the ethics committee of the University of Regensburg (approval 22‐2802_12‐101) and complies with the Declaration of Helsinki. Inclusion criteria were (i) planned LV endomyocardial biopsy to test for ATTR‐CM in patients presenting to our clinic with signs and/or symptoms of heart failure or to rule out AL‐amyloidosis, (ii) age ≥18 years.

**Figure 1:**
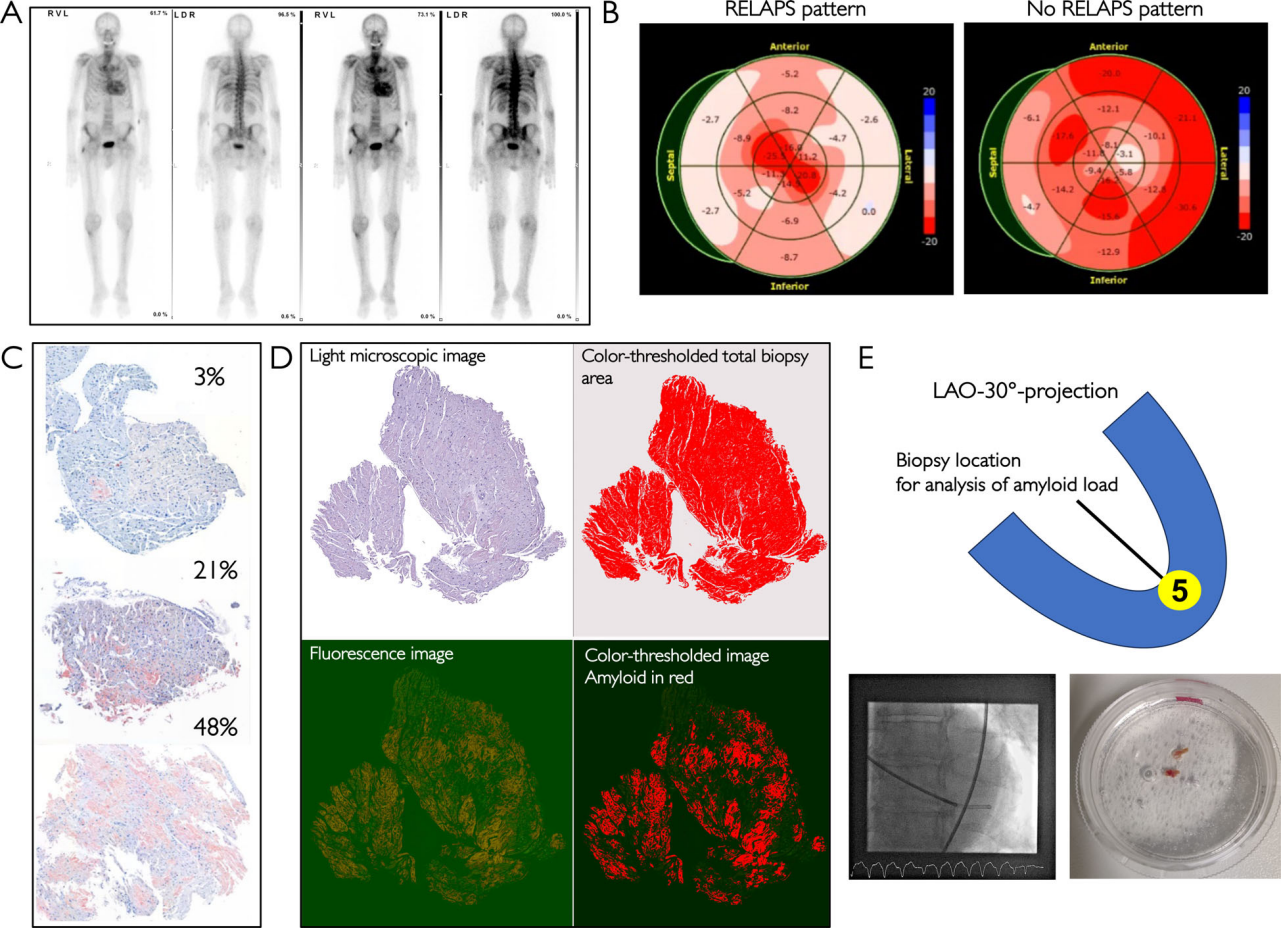
(*A*) Example of study patient with Perugini III bone scintigraphy of TC^99^m‐HDP 3 h post‐injection and with different intensities of nucleotide detection. (*B*) Left panel: bull's eye plot depicting regional peak‐systolic longitudinal strain (%) across the segmented left ventricle of a patient with transthyretin amyloid cardiomyopathy (ATTR‐CM) and positive (calculated) relative apical sparing (RELAPS) pattern. The most pronounced strain reduction is observed in basal and midventricular areas, as highlighted by the lighter red areas. The strain gradient decreases towards the apex with darker red colours, indicating high contractility in the apical regions. Right panel: example of a bull's eye plot for a patient without (calculated) RELAPS pattern. (*C*) Representative histological images showing amyloid deposition in cardiac tissue samples stained with Congo red at different percentages of transthyretin amyloid load. Top panel: minimal amyloid deposition (3% of surface area), with sparse and faintly red‐stained areas in the extracellular matrix, indicating low amyloid content. Mid panel: moderate amyloid deposition, with more prominent and widespread red‐stained areas interspersed throughout the myocardial tissue (21% of surface area). The deposition begins to disrupt the myocardial structure, showing a patchy distribution. Bottom panel: severe amyloid deposition, with extensive and dense red‐stained areas (48% of surface area), covering a significant portion of the tissue. The myocardial architecture is highly compromised, indicating advanced infiltration of amyloid fibrils. (*D*) Illustration of the quantification methodology for amyloid load with the different acquisition channels of the same slide (i.e. upper left: light microscopic image; upper right: colour‐thresholded image for selection of total tissue area; lower left: fluorescence image; lower right: colour thresholded image showing amyloid in red). (*E*) For the analysis of left ventricular amyloid load, we used biopsies that were taken in a left anterior oblique (LAO) 30° projection, targeting the forceps at the 5 o'clock position of the apex (see schematic in the top panel and a fluoroscopic example of catheter placement in the bottom left panel, forceps still in catheter, therefore final placement not visible). Two retrieved left ventricular endomyocardial biopsies can be seen on the bottom right.

Prior diagnostics were based on current recommendations^5^, ^6^ and typically included either MRI or scintigraphy, as well as echocardiography and natriuretic peptides plus troponin. Typical clinical reasons for endomyocardial biopsy in patients with suspected amyloid cardiomyopathy were: (i) Perugini I and II scores in the scintigraphy scan, (ii) monoclonal gammopathy in the immunofixation and/or elevated light chains (regardless of scintigraphy result if echocardiography or MRI indicated amyloid cardiomyopathy), and (iii) MRI indicating amyloid cardiomyopathy with possible differential diagnoses not resolved by laboratory results and/or echocardiography. *Figure* *1A* shows an example of a Perugini III scintigraphy scan.

### Clinical data

Patient data were collected during peri‐interventional admission to the University Hospital Regensburg, Germany.

### Standard echocardiography

Transthoracic echocardiography was performed peri‐interventionally by experienced physicians (Philips Affinity series, Philips, Amsterdam, The Netherlands). Conventional echocardiographic parameters were measured according to recommendations by the German Society of Cardiology.^16^

### Speckle‐tracking imaging and strain analysis

We used standard methodologies for speckle tracking to measure GLS using commercially available software (QLAB 13.0, Philips, Hamburg, Germany). GLS was obtained by averaging all values of segmental peak strain in the three apical views.^17^ Apical sparing was evaluated first by visual impression and then calculated by RELAPS (average apical longitudinal strain/[average basal longitudinal strain + average midventricular longitudinal strain]). Values >1 were considered positive for RELAPS. A visual example of apical sparing (confirmed by calculated RELAPS) is displayed in *Figure* *1B*.

### Left ventricular endomyocardial biopsy

Left ventricular endomyocardial biopsy was performed by experienced operators in the catheter laboratory of the University Hospital Regensburg according to standard of care procedure guidelines. For analysis of LV amyloid load, biopsies were taken in a left anterior oblique 30° projection, targeting the forceps at the 5 o'clock position of the apex (*Figure* *1E*).

### Histology and quantification of amyloid load

Initial histology was performed in our accredited Institute for Pathology (University of Regensburg, Germany). After the presence of amyloid had been confirmed at our centre, LV paraffin blocks were sent to the Institute for Pathology of the University of Kiel, Germany, for central analysis of amyloid load and for disease subtyping (see online supplementary material). Amyloid load was quantified as the percentage of tissue area occupied by amyloid deposits. For patients with >1 biopsy fragment available for quantification, the mean value of amyloid load across all high‐quality fragments was used in analyses to account for intra‐patient variability. Analysis of amyloid load in multiple biopsies from the same patients in a set of patients with >1 apical biopsy available (*n* = 16) demonstrated generally low intra‐patient variability (online supplementary *Figure Appendix* *S1*). Examples of Congo red staining are displayed in *Figure* *1C*. The presented patients display an amyloid load of 3%, 21%, and 48% (top to bottom) of the analysed area. *Figure* *1D* shows the above‐mentioned quantification methodology with the different acquisition channels of the same slide.

### Statistical analysis

Data are shown as mean ± standard error of the mean (SEM) unless indicated otherwise. For normally distributed variables, Student's *t*‐test was used; for ordinal or non‐normal data, the Mann–Whitney test was applied. Group comparisons employed one‐way ANOVA or Kruskal–Wallis tests with appropriate post‐hoc analyses, as detailed in figure legends. Correlations were evaluated by simple linear regression. All analyses were two‐sided and performed in GraphPad Prism 10, with *p* < 0.05 considered statistically significant.

## Results

In our study, we included 61 patients presenting to our clinic with high probability of cardiac amyloidosis for cardiac biopsy to confirm a diagnosis of ATTR‐CM or to rule out cardiac AL‐amyloidosis. Overall, 64.6% of these patients had elevated serum monoclonal light chains type kappa and 56.3% had elevated serum monoclonal light chains type lambda in the serum free light‐chain assay; 18.8% of patients displayed pathological kappa/lambda‐ratios and 22.9% showed monoclonal gammopathy of undetermined significance (MGUS) in the serum protein electrophoresis with immunofixation (SPIE). Patient data for all study patients can be found in *Table* *1*. Of all patients, four patients were subsequently diagnosed with cardiac AL‐amyloidosis based on the histological analysis. One patient had insufficient cardiac material for amyloidosis subtyping and will be receiving additional diagnostics. The four patients with cardiac AL amyloidosis and the uncertain patient were excluded from the following analyses of ATTR patients, thus 56 patients with histologically confirmed ATTR‐CM were analysed per protocol. All patients with ATTR‐CM showed expression of wild‐type alleles of *TTR*.

**Table 1:** Clinical and demographic characteristics of all patients that underwent biopsy

Age (years), mean ± SEM	78.6 ± 0.9
Male sex (%)	82.0
EF (%), mean ± SEM	53.8 ± 1.1
HFpEF (%)	73.3
HFmrEF (%)	21.7
HFrEF (%)	5.0
NYHA class (%)	
I	6.8
II	72.7
III	20.5
IV	0
Pathological monoclonal light chains type kappa (%) (elevated when >22.4 mg/L)	64.6
Pathological monoclonal light chains type lambda (%) (elevated when >27.0 mg/L)	56.3
Pathological kappa/lambda ratios (%) (elevated when ratio >1.65)	18.8
MGUS (%)	22.9
Histologically confirmed TTR amyloidosis (%)	91.8
AL amyloidosis (%)	6.6
Mixed amyloidosis (%)	0
Undetermined form of amyloidosis (%)	1.6
Perugini score (%)	
III	50.0
II	31.3
I	16.7
0	2.1
Biopsies taken per patient (*n*), mean ± SEM	4.2 ± 0.2

AL, light chain; EF, ejection fraction; HFmrEF, heart failure with mildly reduced ejection fraction; HFpEF, heart failure with preserved ejection fraction; HFrEF, heart failure with reduced ejection fraction; MGUS, monoclonal gammopathy of undetermined significance; NYHA, New York Heart Association; SEM, standard error of the mean; TTR, transthyretin.

Of the 61 patients first included, 48 patients (78.7%) had an initial ‘bone tracer’ scintigraphy scan with an assigned Perugini score. Of these 48 patients, 2.1% were categorized as Perugini 0, 16.7% as Perugini 1, 31.3% as Perugini 2%, and 50.0% as Perugini 3. Two of the patients with Perugini 1 were subsequently diagnosed with AL‐amyloidosis based on histology and, as mentioned, were not included in the following analyses. The singular patient with suspected ATTR‐CM and Perugini 0 had an LV amyloid load of 0.4%.

**Figure 2:**
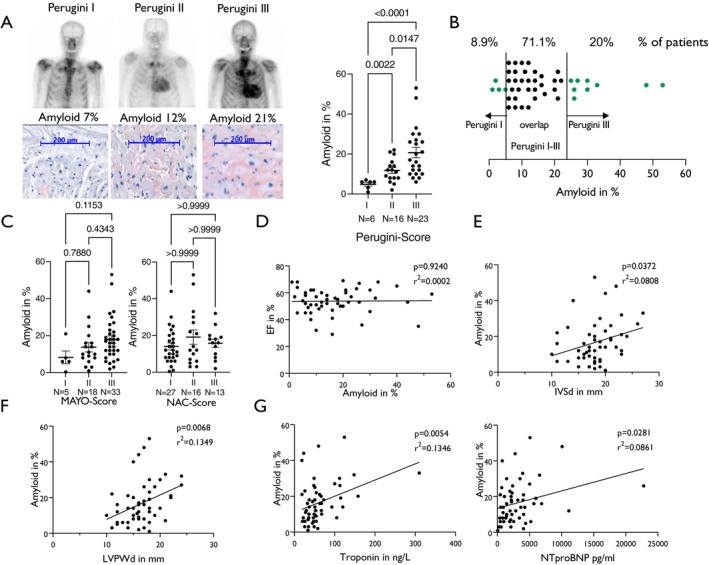
(*A*) Left panel: representative scintigraphy scans of patients with Perugini score I, II, and III with their measured amyloid burden. Representative Congo red staining of left ventricular endomyocardial biopsy tissue sections below. Right panel: mean ± standard error of the mean values of left ventricular amyloid load categorized according to the Perugini score. Brown–Forsythe ANOVA with Dunnett post‐test (overall *p* < 0.0001, post‐test values displayed in the figure). (*B*) Graphical representation of the overlap of left amyloid loads of patients with Perugini scores I‐III; 71.1% of patients were present in an overlap area where the Perugini score could not clearly discriminate amyloid load accurately. (*C*) Mean ± standard error of the mean values of amyloid load categorized according to Mayo (left panel) and National Amyloidosis Centre (NAC) scores (right panel). Left and right panel: Kruskal–Wallis test with Dunn's post‐test (overall *p* = n.s., post‐test values displayed in the figure). (*D*) Correlation by simple linear regression of left ventricular ejection fraction (EF) and left ventricular amyloid load (%). (*E*) Correlation by simple linear regression of interventricular diastolic septum thickness (IVSd in mm) and left ventricular amyloid load (%). (*F*) Correlation by simple linear regression of left ventricular diastolic posterior wall thickness (LVPWd in mm) and left ventricular amyloid load (%). (*G*) Correlation by simple linear regression of high‐sensitivity‐troponin T (left panel) and N‐terminal pro‐B‐type natriuretic peptide (NT‐proBNP) levels (right panel) and left ventricular amyloid load (%).

Left ventricular amyloid load in the apical biopsies of patients with available Perugini score showed a significant increase with increasing Perugini score (*Figure* *2A*). However, in our large cohort, cardiac amyloid load was only specific for a Perugini score of 1 if amyloid load was <6% and specific for a Perugini of 3 if amyloid load was >22% (*Figure* *2B*). This means an overlap between LV amyloid contents and Perugini I–III scores occurred in 71.1% of patients for whom the scintigraphy is unable to accurately define the LV amyloid load (*Figure* *2B*).

We next analysed all 56 patients with histologically confirmed ATTR‐CM.

In these patients, neither the National Amyloidosis Centre (NAC) score nor the Mayo score commonly used for prognostication in ATTR‐CM could clearly discriminate patients with lower and higher amyloid loads at first diagnosis (*Figure* *2C*).

As expected for a disease with mostly preserved ejection fraction, LV ejection fraction and amyloid content did not show a significant correlation (*Figure* *2D*). However, LV septum thickness and posterior wall thickness showed a significant positive linear correlation with the amyloid content in the biopsies (*Figure* *2E*,*F*). Moreover, isolated (i.e. not contained in a risk stratification score) troponin values and—to a lesser extent—N‐terminal pro‐B‐type natriuretic peptide (NT‐proBNP) values also showed significant linear correlation with amyloid content (*Figure* *2G*).

Interestingly, 18.2% of patients with ATTR‐CM were women (data for male vs. female patients can be found in online supplementary *Table Appendix* *S1*). While women were of similar age as male patients (male patients *n* = 46, age 79.3 ± 0.8 years vs. female patients *n* = 10, age 79.2 ± 2.8 years, *p* = n.s.), females showed significantly higher NT‐proBNP at first diagnosis without overt signs of cardiac decompensation, increased rates of atrial fibrillation, or increased septal or posterior wall thickness (online supplementary *Table Appendix* *S1*). Notably, while not statistically significant (possibly due to the small sample size in the female cohort and therefore lack of power), female patients showed a trend towards higher LV amyloid load in the biopsies at first diagnosis (females: mean = 18.6 ± 4.0% vs. males: 15.3 ± 1.7%, *p* = n.s.), potentially indicating that female patients may have been diagnosed at a later stage of the disease.

Forty‐eight patients had an echocardiography scan with sufficient quality for strain imaging. We analysed LV GLS, defined ‘bull's‐eye’ plots for these patients (*Figure* *1B*) and calculated RELAPS. Baseline characteristics for these patients, stratified by the presence or absence of RELAPS can be found in *Table* *2*.

**Table 2:** Patients with histologically confirmed transthyretin amyloid cardiomyopathy and strainable echocardiography

Patient data	All ATTR patients	No relative apical sparing pattern (*n* = 26)	Relative apical sparing pattern (*n* = 22)	*p*‐value
Age (years), mean ± SEM	79.3 ± 0.8	80.1 ± 1.2	79.0 ± 1.1	0.1975
Male sex (%)	82.1	88.5	81.8	>0.9999
EF (%), mean ± SEM	53.8 ± 1.3	56.46 ± 1.4	50.8 ± 2.2	**0.0380**
Phenotypic HFpEF (%)	75.0	80.8	72.7	0.5048
Phenotypic HFmrEF (%)	19.6	19.2	22.7	>0.9999
Phenotypic HFrEF (%)	5.4	0	4.6	0.2048
NYHA class				
I	7.9	14.3	0	0.2394
II	71.1	66.7	75.0	0.7711
III	21.0	19.0	25.0	>0.9999
Atrial fibrillation (%)	62.5	42.3	72.7	**0.0199**
aHTN (%)	80.4	92.3	68.2	0.1194
Diabetes (%)	25.0	23.1	22.7	>0.9999
CKD (%)	48.2	50.0	45.5	0.7799
eGFR (ml/min), mean ± SEM	55.5 ± 2.3	58.0 ± 3.7	54.8 ± 2.9	0.5062
Creatinine (mg/dl), mean ± SEM	1.3 ± 0.1	1.2 ± 0.1	1.3 ± 0.1	0.8891
MGUS (%)	17.9	34.6	4.5	**0.0134**

aHTN, arterial hypertension; ATTR, transthyretin amyloidosis; CKD, chronic kidney disease; EF, ejection fraction; HFmrEF, heart failure with mildly reduced ejection fraction; HFpEF, heart failure with preserved ejection fraction; HFrEF, heart failure with reduced ejection fraction; NYHA, New York Heart Association; eGFR, estimated glomerular filtration rate; MGUS, monoclonal gammopathy of undetermined significance; SEM, standard error of the mean.

**Figure 3:**
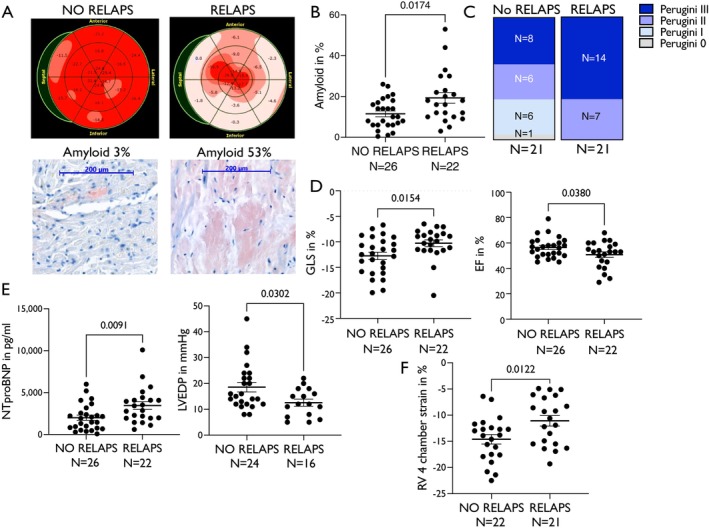
(*A*) Representative bull's eye plots of left ventricular strain studies of patients without relative apical sparing (RELAPS) pattern (left panel) and with RELAPS (right panel) and their assessed left ventricular amyloid load with representative Congo red staining from the same patients below. (*B*) Mean ± standard error of the mean of left ventricular amyloid load of patients with or without RELAPS. Mann–Whitney test. (*C*) Comparison of Perugini scores of patients with RELAPS and without RELAPS (if scintigraphy scan available). Patients with RELAPS displayed higher Perugini scores. (*D*) Mean ± standard error of the mean of global longitudinal strain (GLS) values (left panel) and ejection fraction (EF) (right panel) stratified according to the presence or absence of RELAPS. Student's *t*‐test. (*E*) Mean ± standard error of the mean of N‐terminal pro‐B‐type natriuretic peptide (NT‐proBNP) values (left panel) and left ventricular end‐diastolic pressure (LVEDP) categorized according to the presence or absence of RELAPS (assessed invasively during biopsy, right panel). Patients with RELAPS displayed higher levels of NT‐proBNP but lower LVEDP. Mann–Whitney test. (*F*) Comparison of patients with and without RELAPS regarding their right ventricular (RV) four‐chamber view strain. Patients with RELAPS displayed lower (i.e. worse) RV strain. Student's *t*‐test.

Twenty‐two patients showed apical sparing concurrent with RELAPS, while 26 did not. Importantly, patients with apical sparing and positive RELAPS (i.e. RELAPS values >1,^14^, ^18^) had significantly higher amyloid load in their LV biopsies (original bull's‐eye plots and histology in *Figure* *3A*, mean data for LV amyloid load in patients with or without RELAPS in *Figure* *3B*). Interestingly, patients with or without RELAPS were of similar age, had similar septal and posterior wall thickness as well as left LV end‐diastolic diameters and estimated glomerular filtration rate (online supplementary *Figure* *S2A–E*). Concurrent with our data showing increased amyloid load with increasing Perugini scores (*Figure* *2A*), patients with apical sparing and available scintigraphy scan had, on average, higher Perugini scores (*Figure* *3C*). Importantly, patients with RELAPS also showed worse overall GLS and ejection fraction (*Figure* *3D*), increased NT‐proBNP (*Figure* *3E*), but, interestingly, lower LV end‐diastolic pressure (LVEDP) (*Figure* *3E*, assessed invasively via LV catheter during the biopsy), which might indicate that apical sparing/RELAPS is a sign of advanced disease with increased stiffness at first diagnosis. However, patients with RELAPS also had significant higher doses of oral diuretics (data not shown), which might also affect LVEDP (but then again showed higher average NT‐proBNP values). Finally, global right ventricular strain (assessed in four‐chamber view) was also worse in patients displaying RELAPS (*Figure* *3F*).

**Figure 4:**
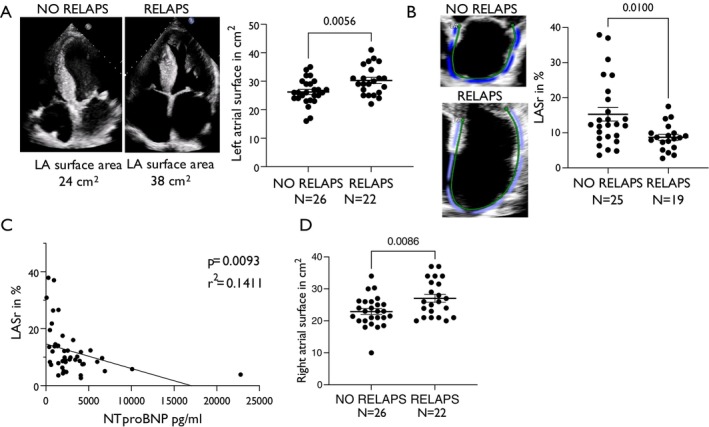
(*A*) Left panel: original registrations of planimetric assessment of left atrial (LA) surface area of patients without and patients with relative apical sparing (RELAPS) pattern. Right panel: mean ± standard error of the mean of LA surface area (in cm^2^) of patients without RELAPS and patients with RELAPS. Student's *t*‐test. (*B*) Original echocardiography recordings (left panel) and mean ± standard error of the mean values (right panel) of left atrial reservoir strain (LASr) of patients without RELAPS and patients with RELAPS. Mann–Whitney test. (*C*) Correlation by simple linear regression of LASr and ‐terminal pro‐B‐type natriuretic peptide (NT‐proBNP) in patients with transthyretin amyloid cardiomyopathy. (*D*) Mean ± standard error of the mean values of right atrial surface area stratified by the presence or absence of RELAPS. Student's *t*‐test.

Notably, in our study population, patients with RELAPS displayed a significantly higher percentage of atrial fibrillation compared to patients without RELAPS (RELAPS vs. no RELAPS: 72.7% vs. 42.3%, *p* = 0.02). Moreover, we found that patients with RELAPS had increased left atrial area compared to patients with no RELAPS pattern, indicating structural remodelling (*Figure* *4A*). However, patients with RELAPS also showed disturbed left atrial strain during the reservoir phase (*Figure* *4B*), which correlated with increased NT‐proBNP (*Figure* *4C*), therefore indicating impaired atrial compliance. Notably, patients with RELAPS also had advanced right atrial remodelling with increased right atrial area at first diagnosis (*Figure* *4D*).

## Discussion

This study investigates the clinical, histological, and echocardiographic differences of patients with histologically confirmed ATTR‐CM, providing insights into the role of apical sparing as a potential marker for disease severity. Our findings support the hypothesis that a RELAPS pattern is associated with advanced stages of cardiac involvement in ATTR‐CM. Our study also shows low overall discriminatory power of the commonly used bone scintigraphy scans to discern cardiac amyloid load based on the Perugini score.

The scintigraphy scans were a reliable tool for the diagnosis of ATTR‐CM. Notably, patients on average showed increasing LV amyloid loads in the biopsies with increasing Perugini scores. However, the overlap between histologically assessed amyloid load and Perugini scores I‐III was large, where the scintigraphy failed to clearly discriminate patients with LV TTR loads between 6% and 22%. Moreover, patients with a Perugini score of III had LV amyloid loads ranging from 6% to 53%. Hence, Perugini scores do not clearly discern patients with moderate and more advanced disease. However, echocardiographic parameters such as septal and posterior wall thickness, as well as troponin and NT‐proBNP values correlated with LV amyloid load, underscoring their importance in the assessment of the disease severity.

Notably, few patients showed LV amyloid loads above ~33%. As the reason for the biopsy is to establish a diagnosis of ATTR‐CM, this could mean that most patients are below 33% amyloid load when their disease first becomes symptomatic. Alternatively, this could also mean that a threshold exists, after which mortality suddenly increases or morbidity prevents access to the healthcare system, therefore limiting the number of patients with very high amyloid loads in their biopsies.

In our analysis, patients with a RELAPS pattern demonstrated significantly higher amyloid load in the LV biopsies. On the other hand, >50% of the analysed patients did not show a RELAPS pattern, demonstrating that its absence clearly does not exclude significant ATTR‐CM. The association of the presence of RELAPS not only with increased amyloid load, but also with lower ejection fraction and elevated NT‐proBNP indicates that RELAPS is a sign of advanced disease, where urgent therapeutic intervention may be of utmost importance. Interestingly, patients with RELAPS showed not only higher amyloid burden, worse GLS and ejection fraction, and higher NT‐proBNP, but paradoxically also lower LVEDP. This may reflect a phenotype of advanced myocardial stiffness and impaired compliance, where wall stress is increased despite reduced filling pressures. In addition, the higher diuretic doses in RELAPS patients may have contributed to the observed lower LVEDP.

Elevated NT‐proBNP reflects increased myocardial wall stress, while elevated troponin indicates myocardial injury, both of which are hallmarks of advanced disease and correlate with LV amyloid load.^19^ However, these scoring systems do not directly quantify or discriminate the actual myocardial amyloid load. It is therefore perhaps not unexpected that patients with impaired renal function, who consequently present with an increased NAC score, demonstrate worse outcomes.^19^, ^20^ Yet, this adverse prognosis may reflect the systemic impact of renal dysfunction rather than solely indicating more advanced cardiac amyloid involvement. We therefore propose that histological assessment of amyloid load and GLS may complement existing staging systems, adding value in the diagnostic and prognostic assessment of cardiac amyloidosis, particularly in patients with wild‐type disease.

Reduced GLS in patients with apical sparing reinforces the utility of strain imaging as a sensitive marker for identifying functional impairment in cardiac amyloidosis^21^ even when LV ejection fraction may (not yet) be impaired.^11^, ^15^

No significant differences were observed between the sparing and non‐sparing groups in age or LV diameter. This suggests that traditional demographic and echocardiographic measures may underestimate the severity of ATTR‐CM. Apical sparing, as quantified by echocardiographic, may reflect the progression of amyloid deposition and provide insights into the stage of the disease. In our study, apical biopsies from patients with apical sparing still showed considerable amyloid burden, supporting the hypothesis that sparing reflects advanced disease, as even the ‘spared’ region is heavily infiltrated. Since biopsies were only taken apically, we cannot assess potential base‐to‐apex gradients, which will require confirmation in future studies using postmortem analyses or intra‐individual biopsies from different ventricular sites. Another limitation could be sampling errors due to the patchy nature of myocardial amyloid deposition, as Congo red staining of different biopsy fragments from the same patient may yield variable loads and staining quality. However, our analysis of intra‐patient variability of amyloid load showed generally low intra‐patient variability. In our study one–two samples were enough to detect even small amounts of amyloid, but clinical practice usually requires more tissue to exclude other diagnoses. We recommend at least three–four biopsies to enable simultaneous amyloid quantification, standard histological staining, and electron microscopy. Atrial amyloid deposits are a risk factor for atrial fibrillation.^22^ It is unknown whether apical sparing may contribute to the development of atrial fibrillation or—taking our data on LV amyloid load into account—potentially also indicates increased atrial TTR load. In our study, patients with RELAPS displayed higher percentages of atrial fibrillation and echocardiographic as well biochemical parameters indicating atriopathy.

While our study provides important validations of LV TTR load and the importance of RELAPS, its single‐centre setting may limit generalizability. Future research should focus on even larger, multicentre cohorts to validate these findings. Furthermore, we only included wild‐type ATTR‐CM patients in our analysis. Therefore, the results might not be generalizable to patients with hereditary amyloidosis, especially considering the large number of *TTR* mutations and the variable cardiac and neurological phenotype of hereditary ATTR‐CM. Longitudinal analyses exploring the prognostic value of LV amyloid load and apical sparing for mortality and response to treatment would further enhance our pathophysiological understanding of ATTR‐CM, but—given effective TTR stabilizers and future therapeutic approaches—the time for these studies may be slipping. Additionally, in our cohort, the proportion of female patients was rather low, which may limit the generalizability of our findings to women with ATTR‐CM. Our exploratory analysis suggested that female patients may present with higher cardiac amyloid loads at first diagnosis, but the small sample size precludes firm conclusions. Larger studies with greater female representation are required to confirm these observations and to explore additional sex‐specific differences. Finally, in our cohort, MGUS was more common in patients without RELAPS (34.6% vs. 4.5%). This difference could partly be explained by diagnostic pathways: patients with MGUS, detected by immunofixation or abnormal light chains, are often referred for endomyocardial biopsy at earlier stages to rule out AL‐amyloidosis. Consequently, the non‐RELAPS group might have been enriched with earlier‐stage patients carrying MGUS.

In conclusion, LV amyloid load correlates with echocardiographic and laboratory marker of advanced disease. RELAPS is associated with increased LV amyloid load, elevated cardiac biomarkers, and reduced myocardial strain at first diagnosis, reinforcing its role as a marker of advanced disease in ATTR‐CM. Our findings underscore the clinical utility of RELAPS and histologically assessed amyloid burden in risk stratification and may inform therapeutic decision‐making in this challenging patient population.

## Supporting information


**Appendix S1.** Supporting Information.
