# Supplementary material for: Left ventricular transthyretin amyloid load and apical sparing in patients with newly confirmed transthyretin amyloid cardiomyopathy
Source: Eur J Heart Fail. 2025 Oct 30;27(12):2979–89. doi: 10.1002/ejhf.70077 (PMC12803544; doi:10.1002/ejhf.70077)
Supplement: Supplementary file 1 — Appendix S1. Supporting Information. [file EJHF-27-2979-s001.zip › ejhf70077-sup-0001-Supinfo.docx]

**Online supplement**

Left ventricular transthyretin amyloid load and apical sparing in patients with newly confirmed transthyretin amyloid cardiomyopathy

Thomas Krammer^1^, Maria J. Baier^1^, Vanessa Lutz^1^, Anna-Christina Hübner^1^, Tilman Zschiedrich^1^, David Lukas^1^, Christian Le Phu^1^, Matthias Wolf^1^, Claire Maassen^1^, Michael Wester^1^, Stefan Neef^1^, Christian Schach^1^, Can-Martin Sag^1^, Katja Evert^2^, Michael Paulus^1^, Christine Meindl^1^, Maria Tafelmeier^1^, Kurt Debl^1^, Lars S. Maier^1^, Stefan Wagner^1^, Christoph Röcken^3^, Julian Mustroph^1,4^

^1^Dept. of Internal Medicine II, University Medical Center Regensburg, Germany

^2^Institute for Pathology, University of Regensburg, Germany

^3^Institute for Pathology, University of Kiel, Germany

^4^Dept. of Pharmacology, University of Regensburg, Germany

**Supplemental methods**

**Study protocol**

The study was approved by the ethics committee of the University of Regensburg (ethics approval 22-2802_12-101). Inclusion criteria were 1) planned left ventricular endomyocardial biopsy to test for transthyretin amyloid cardiomyopathy (ATTR-CM) or to rule out AL-amyloidosis, 2) age ≥ 18 years. In this observational study, we prospectively enrolled 61 patients presenting to our clinic with high probability of cardiac amyloidosis between May 2022 and May 2024.

Contraindications for study inclusion were 1) inability to understand the informed consent / incapacitated patients (unless the legal guardian consented to study participation) 2) clinical contraindications for endomyocardial biopsy and patients in whom a myocardial biopsy was considered excessively risky for technical reasons (e.g. echocardiographically thinned myocardium, inability to perform a pericardiocentesis due to anatomical obstacles, complex vascular anomalies in the access route to the heart) and 3) patients with infectious diseases with known cardiac involvement for whom sample processing requires a safety level >S2 (German standard). Of note, the current standard approach for endomyocardial biopsy in our center is left ventricular biopsy due to the – in our experience – superior safety profile of left ventricular biopsy, which is also reflected in a low number of complications in large studies investigating the biopsy methodology(1). Also, left ventricular biopsy enables simultaneous angiography of the coronary arteries in a collective mostly screened by scintigraphy.

Left ventricular endomyocardial biopsy was performed in patients presenting to our clinic with signs and/or symptoms of heart failure and suspected ATTR-CM or in patients requiring exclusion of AL amyloidosis. Prior diagnostics were based on current recommendations(2, 3) and for suspected ATTR-CM typically included either MRI or scintigraphy, as well as echocardiography, and natriuretic peptides plus troponin. Diagnostics were indicated and performed either by external physicians (cardiologists, general practitioners, nuclear medicine physicians) or within our university facility (mostly outpatients, some inpatients). To discern light-chain and TTR amyloid cardiomyopathy, light chains analysis and immunofixation were performed in blood samples and urine.

Typical clinical reasons for endomyocardial biopsy in patients with suspected amyloid cardiomyopathy were: **1)** Perugini I and II scores in the scintigraphy scan, **2)** monoclonal gammopathy in the immunofixation and/or elevated light chains (regardless of scintigraphy result if echocardiography or MRI indicated amyloid cardiomyopathy), and **3)** MRI indicating amyloid cardiomyopathy with possible differential diagnoses not resolved by laboratory results and/or echocardiography. Endomyocardial biopsy was also offered to some patients with Perugini III score if uncertainty regarding (additional/differential) diagnoses persisted or if diagnostic certainty was requested by patients and/or health care providers. **Figure 1A** shows an example of a Perugini III scintigraphy scan.

**Clinical data**

Patient data was collected during periinterventional inpatient admission at the Department of Internal Medicine II of the University Hospital of Regensburg, Germany, after written informed consent had been ascertained. Clinical parameters included comorbidities, medical history, medication, vital signs and bloodwork. Additionally, external results such as scintigraphy reports from other hospitals or health care providers were collected. All types of atrial fibrillation (AF) were defined as atrial fibrillation independent from duration (e.g. paroxysmal, persistent and permanent AF). Coronary artery disease was defined as a history of myocardial infarction, angioplasty, or angiographically documented coronary artery stenosis fulfilling current guideline recommendations(4).

**Standard echocardiography**

Transthoracic echocardiography was performed periinterventionally by experienced physicians (Philips Affinity series, Philips, Amsterdam, Netherlands). Echocardiographic recordings were digitally recorded and made available for offline analysis. Conventional echocardiographic parameters were measured according to the recommendations of the German Society of Cardiology(5). While all physicians were aware of the suspected amyloid cardiomyopathy due to clinical and logistical reasons, all results were acquired and reports finalized before (in parallel) the histology report was obtained and sonographers were unaffiliated with the study data analysis.

**Speckle tracking imaging and strain analysis**

We used standard methodologies for speckle tracking to measure global longitudinal strain (GLS) using commercially available software (QLAB 13.0, Philips, Hamburg, Germany). The investigators were blinded upon analysis of RELAPS with regards to the amyloid content and the type of amyloid cardiomyopathy (i.e. wildtype ATTR or AL amyloidosis). After automated tracing of the LV endocardial border, the software automatically tracked the entire myocardium throughout the cardiac cycle in the apical four-chamber, two-chamber, and three-chamber views. If automated tracking was subpar, manual adjustments were made by the central investigators. GLS was obtained by averaging all values of segmental peak strain in the three apical views.(6) Technically inadequate strain recordings of GLS were adjudicated by two blinded, independent and experienced sonographers and consequently excluded (n=8). Apical sparing was evaluated first by visual impression of experienced sonographers (“eye balling”) and then calculated by RELAPS (average apical longitudinal strain / [average basal longitudinal strain + average midventricular longitudinal strain]). A RELAPS value > 1 was considered positive for relative apical sparing. A visual example of apical sparing (confirmed by calculated RELAPS) is displayed in **Figure 1B left panel**.

**Left ventricular endomyocardial biopsy**

Left ventricular endomyocardial biopsy was performed by experienced operators in the catheter laboratory of the University Hospital of Regensburg. The procedure was performed according to standard of care procedure guidelines. Patients were typically either biopsied via the right femoral artery access or the right radial access. Typically, an 8F femoral access was used to first perform a coronary angiogram (using standard diagnostic catheters in 5F-6F for diagnostics, followed by appropriate interventional catheters if deemed necessary). After the diagnostic angiogram (± coronary intervention; n=9), a 5F pig-tail catheter sheathed into an 8F MPA-catheter was advanced through the aortic valve and was subsequently used to advance the 8F MPA into the left ventricle. Then, after application of a small bolus of contrast agent and subsequent verification that no papillary muscle was affected by catheter placement, biopsies were taken using a 2.2 mm biopsy forceps (Fehling Instruments). Radial procedure was similar, but a 7F MPA and a 1.8 mm biopsy forceps (Fehling Instruments) were used instead. Typically, 4-5 clinical biopsies were taken, of which one biopsy was fixated in Karnovsky fixative for electron microscopy if uncertainties regarding the etiology of the disease persisted after histological analysis of the formalin-fixed samples. For the analysis of left ventricular amyloid load for this publication, we used biopsies that were taken in a left anterior oblique (LAO) 30° projection, targeting the forceps at the 5 ‘o clock position of the apex (see **Figure 1E** for a schematic [top], a fluoroscopic example of catheter placement [bottom left, forceps still in catheter, therefore final placement not visible] and two retrieved left ventricular endomyocardial biopsies [bottom right], some variability due to patient placement and anatomical differences of the heart of course is to be expected and therefore cannot be excluded). In the 61 patients included into our study, no embolism or stroke of any kind occurred and no patients died. Two patients had pericardial effusion requiring pericardiocentesis which extended the hospital stay for clinical monitoring. Both showed complete recovery after one, respective two days.

**Histology and quantification of amyloid load**

Initial histology was performed in our accredited Institute for Pathology (University of Regensburg, Germany). All samples had been fixed in formalin and embedded in paraffin (FFPE). Serial sections were cut from each paraffin block and stained with standard dyes including hematoxylin and eosin (H&E), Congo red, and Elastica van Gieson. The presence of amyloid was confirmed when a typical green-yellow-orange birefringence was found in cross-polarized light in Congo red stained tissue sections using a polarization microscope (Nikon ECLIPSE Ci POL; Hamburg, Germany). After the presence of amyloid had been confirmed by experienced pathologists at our center, paraffin blocks of embedded left ventricular tissue were sent to the Institute for Pathology of the University of Kiel, Germany, for central analysis of the amyloid load and for subtyping of the disease. All staff at the Kiel facility were unaware of echocardiography data but for clinical reasons received patient information on a case-by-case if required to reach a diagnosis. Classification of amyloid was done as described elsewhere(7).

The amyloid load of a tissue section was calculated by dividing an area containing amyloid deposits by the total tissue area, resulting in a ratio of 0 to 100%. To determine

both areas, Congo red stained tissue slides were scanned in consecutive bright field

and fluorescence mode using a Hamamatsu NanoZoomer 2.0 RS scanner (Hamamatsu Photonics Deutschland GmbH, Herrsching am Ammersee, Germany) with fluorescence option. The bright field image was used to count the pixels comprising total tissue by applying the color threshold function of ImageJ, version 1.52p(8) and adjusting brightness and saturation values until all tissue areas were selected. Fluorescence of Congo red was visualized in the Alexa Fluor 555 nm channel with an excitation wavelength of 560 nm and an emission wavelength of 607 nm, appearing yellow orange. This channel also showed an undesirable diffuse autofluorescence of the whole tissue. To compensate for this, tissue sections were also scanned in the Alexa Fluor 488 channel with excitation wavelength of 485 nm and emission wavelength of 525 nm, appearing green. This channel showed only autofluorescence of the tissue, but no signal from Congo red. In the overlay of both images, Congo red appeared yellow orange to orange, while background and non-amyloid tissue appeared green to yellow green. The color threshold function of Image J was used to select all pixels showing amyloid, this time by adjusting the hue value. Sections were scanned at 400x magnification and final images analyzed with ImageJ. For patients with >1 biopsy fragment available for quantification, the mean value of amyloid load across all high-quality fragments was used in analyses to account for intra-patient variability. Analysis of amyloid load in multiple biopsies from the same patients in a set of patients with >1 apical biopsy available (n=16) demonstrated generally low intra-patient variability (**Supplementary Figure 1**). Examples of Congo red stainings from patients with different amyloid burden are displayed in **Figure 1C**. The presented patients display an amyloid burden of 3%, 21% and 48% (top to bottom) of the analyzed area. **Figure 1D** shows the above-mentioned quantification methodology with the different acquisition channels of the same slide (i.e. upper left: light microscopic image, upper right: color-thresholded image for selection of total tissue area, lower left: fluorescence image, lower right: color thresholded image showing amyloid in red).

**Supplemental tables**

**Supplemental table 1: Data for female and male ATTR-CM patients**

| **Sex** | **Female ATTR-CM patients (N= 10)** | **Male ATTR-CM patients (N=46)** | **p-values** |
| --- | --- | --- | --- |
| **Age (years ± SEM)** | 79.20 ± 2.82 | 79.33 ± 0.84 | 0.9548 |
| **Ejection fraction (mean ± SEM)** | 48.78 ± 3.37 | 54.86 ± 1.22 | 0.0522 |
| **NTproBNP (mean ± SEM)** | 5204 ± 1063 | 2807 ± 503 | p=0.0488 |
| **Atrial fibrillation (%)** | 40.00 | 63.04 | 0.0961 |
| **Left ventricular amyloid load** | 18.6 ± 4.0 | 15.3 ± 1.7 | 0.4100 |
| **Reported dyspnea at presentation (%)** | 100 | 78.26 | 0.1792 |
| **Presence of peripheral edema at presentation (%)** | 20 | 32.61 | 0.7055 |
| **IVSd (mean ± SEM)** | 18.00 ± 1.33 | 18.14 ± 0.53 | 0.5928 |
| **LVPWd (mean ± SEM)** | 16.78 ± 1.19 | 16.33 ± 0.47 | 0.7072 |
| **Perugini III (%)** | 85.72 | 46.15 | 0.1234 |
| **Perugini II (%)** | 14.28 | 35.90 | 0.3083 |
| **Perugini I (%)** | 0 | 15.39 | 0.5732 |
| **Perugini 0 (%)** | 0 | 2.56 | >0.9999 |

**Supplemental table 1.:** Clinical and demographic characteristics of female and male ATTR-CM patients. Abbreviations: EF: ejection fraction; AF: atrial fibrillation; IVSd: diastolic interventricular septum thickness; LVPWd: left ventricular diastolic posterior wall thickness; SEM: standard error of the mean. Percentages are provided for categorical variables (e.g. sex).

**Supplementary figure legends**

**Supplementary figure 1:**

Quantitative amyloid load per patient based on apical endomyocardial biopsies in a set of patients with more than one biopsy available for quantification. Mean amyloid load is shown in % per patient, with intra-patient standard error of mean (±SEM). The analysis demonstrated a range of standard error of the mean (SEM) values from 0.25 to 7.86, indicating that while some patients showed highly consistent results across samples, others displayed some variability, reflecting the patchy distribution of amyloid.

**Supplementary figure 2:**

Dot plots displaying individual data points for clinical and laboratory parameters comparing patients with and without relative apical sparing (RELAPS). Each plot includes mean ± SEM and corresponding p-values for group comparisons. **A)** Age: Data points range across a broad spectrum of years, with no significant difference indicated. Mann-Whitney-test. **B)** Interventricular diastolic septum thickness (IVSDd): septum thickness measurements are distributed between ~10 mm and ~25 mm in both groups. Student’s t-test. **C)** Left ventricular diastolic posterior wall thickness (LVPWd) values range from ~5 mm to ~20 mm, with no significant difference noted. Student’s t-test. **D)** Left ventricular enddiastolic diameter (LVEDD): measurements of LVEDD are distributed between ~30 mm and ~60 mm. Student’s t-test. **E)** Estimated glomerular filtration rate (GFR): GFR percentages range from ~20% to ~100% in both groups. Student’s t-test.

**Supplemental references**

1. Chimenti C, Frustaci A. Contribution and Risks of Left Ventricular Endomyocardial Biopsy in Patients With Cardiomyopathies. *Circulation*. 2013;**128**(14):1531-1541.

2. Garcia-Pavia P, Rapezzi C, Adler Y, Arad M, Basso C, Brucato A, et al. Diagnosis and treatment of cardiac amyloidosis: a position statement of the ESC Working Group on Myocardial and Pericardial Diseases. *Eur Heart J*. 2021 Apr 21;**42**(16):1554-1568.

3. Arbelo E, Protonotarios A, Gimeno JR, Arbustini E, Barriales-Villa R, Basso C, et al. 2023 ESC Guidelines for the management of cardiomyopathies: Developed by the task force on the management of cardiomyopathies of the European Society of Cardiology (ESC). *European Heart Journal*. 2023;**44**(37):3503-3626.

4. Vrints C, Andreotti F, Koskinas KC, Rossello X, Adamo M, Ainslie J, et al. 2024 ESC Guidelines for the management of chronic coronary syndromes. *Eur Heart J*. 2024 Sep 29;**45**(36):3415-3537.

5. Hagendorff A, Fehske W, Flachskampf FA, Helfen A, Kreidel F, Kruck S, et al. Manual zur Indikation und Durchführung der Echokardiographie – Update 2020 der Deutschen Gesellschaft für Kardiologie. *Der Kardiologe*. 2020;**14**(5):396-431.

6. Voigt JU, Pedrizzetti G, Lysyansky P, Marwick TH, Houle H, Baumann R, et al. Definitions for a common standard for 2D speckle tracking echocardiography: consensus document of the EACVI/ASE/Industry Task Force to standardize deformation imaging. *J Am Soc Echocardiogr*. 2015 Feb;**28**(2):183-193.

7. Schönland SO, Hegenbart U, Bochtler T, Mangatter A, Hansberg M, Ho AD, et al. Immunohistochemistry in the classification of systemic forms of amyloidosis: a systematic investigation of 117 patients. *Blood*. 2012;**119**(2):488-493.

8. Schneider CA, Rasband WS, Eliceiri KW. NIH Image to ImageJ: 25 years of image analysis. *Nat Methods*. 2012 Jul;**9**(7):671-675.
