# Supplementary figures and images for: Left ventricular transthyretin amyloid load and apical sparing in patients with newly confirmed transthyretin amyloid cardiomyopathy
Source: Eur J Heart Fail. 2025 Oct 30;27(12):2979–89. doi: 10.1002/ejhf.70077 (PMC12803544; doi:10.1002/ejhf.70077)

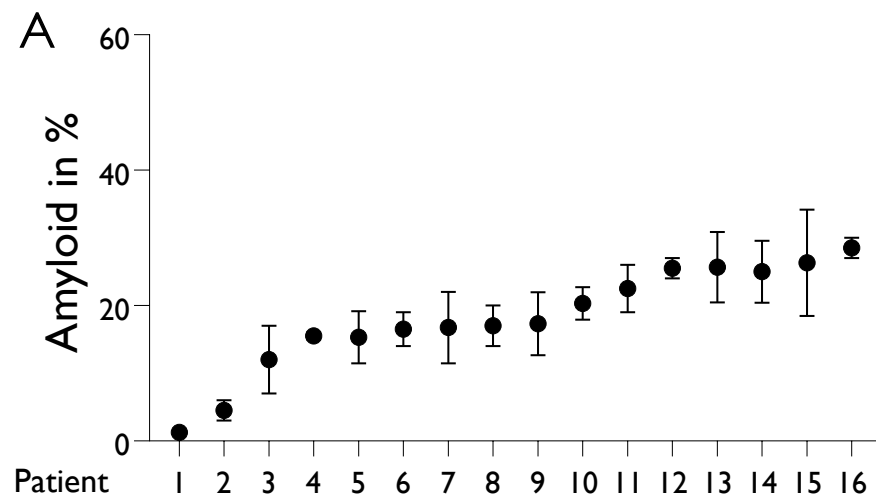

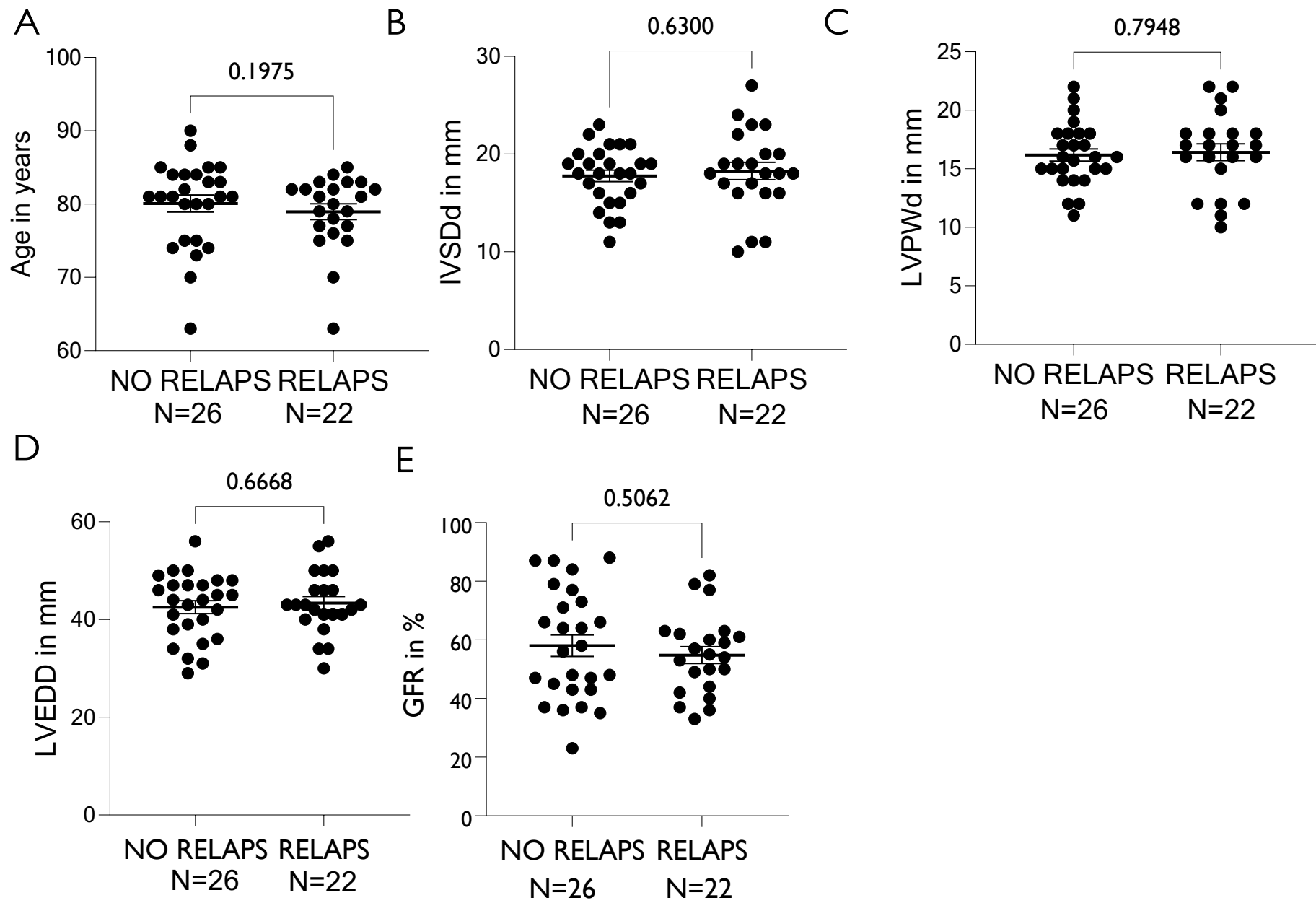

Supplementary figure 2

Supplement: Supplementary file 1 — Appendix S1. Supporting Information. [file EJHF-27-2979-s001.zip › supp_figures_krammeretal2025-2.pdf]
